# A complete ancient RNA genome: identification, reconstruction and evolutionary history of archaeological Barley Stripe Mosaic Virus

**DOI:** 10.1038/srep04003

**Published:** 2014-02-06

**Authors:** Oliver Smith, Alan Clapham, Pam Rose, Yuan Liu, Jun Wang, Robin G. Allaby

**Affiliations:** 1 School of Life Sciences, Gibbet Hill Campus, University of Warwick, Coventry CV4 7AL; 2 The Austrian Archaeological Institute; Cairo Branch, Zamalek, Sharia Ismail Muhammed, Apt 62/72, Cairo, Egypt; 3 BGI-Europe-UK, 9 Devonshire Square, London, EC2M 4YF, UK; 4 BGI-Shenzhen, Shenzhen 518083, China

## Abstract

The origins of many plant diseases appear to be recent and associated with the rise of domestication, the spread of agriculture or recent global movements of crops. Distinguishing between these possibilities is problematic because of the difficulty of determining rates of molecular evolution over short time frames. Heterochronous approaches using recent and historical samples show that plant viruses exhibit highly variable and often rapid rates of molecular evolution. The accuracy of estimated evolution rates and age of origin can be greatly improved with the inclusion of older molecular data from archaeological material. Here we present the first reconstruction of an archaeological RNA genome, which is of Barley Stripe Mosaic Virus (BSMV) isolated from barley grain ~750 years of age. Phylogenetic analysis of BSMV that includes this genome indicates the divergence of BSMV and its closest relative prior to this time, most likely around 2000 years ago. However, exclusion of the archaeological data results in an apparently much more recent origin of the virus that postdates even the archaeological sample. We conclude that this viral lineage originated in the Near East or North Africa, and spread to North America and East Asia with their hosts along historical trade routes.

The evolution of domestication is associated with the rise of many diseases attributed to an agricultural origin^1^. The unprecedented population densities of humans, domesticated animals and plants in which efficient transmission rates were possible provided new pools for disease. This combined with the novel juxtaposition of species as domesticates came into contact with humans, each other and indigenous wild species in new environments facilitated the transfer of diseases between species, often with an associated increased virulence in the adopted host. While this process has long been appreciated as an origin of many human diseases^1^, more recently it has become apparent that the origins of many domesticated plant diseases are recent, and can be categorized into three principal time periods of origin^2^,^3^,^4^. Firstly, many diseases may be associated with the origins of agriculture as increased plant stand densities and intensifying agricultural practices caused both the amplification of existing diseases from the wild progenitors as well as transmission from other wild species of the centre of crop origin for crops such as wheat, maize and rice^2^,^3^,^4^,^5^,^6^,^7^,^8^,^9^. Secondly, subsequent to domestication, the spread to new environments with agricultural expansion caused domesticated plants to come into contact with new indigenous wild populations resulting in host transfer to crops in the past few thousand years such as the transfer of pathogens from wild European orchids to Brassicas 1,000 years ago^10^,^11^. Thirdly, more recent global movements of plants and disease vectors in the past few hundred years have also caused the emergence of significant pathogens from wild hosts from quite disparate geographies, such as the Israeli origin of Tomato yellow leaf virus which adopted tomato, an Andean crop, as a host that subsequently spread to the Americas, Japan and Australia^2^.

Emerging infectious diseases of plants are recognized as a growing threat to global food security, and of those viruses account for almost half^12^. Therefore a better understanding of the origins of diseases is of significant importance to the management of global food resources. Ancient DNA and RNA of viruses obtained directly from herbaria and long-term field sampling have demonstrated that heterochronous sampling serves to improve phylogenetic based estimates by countering recent calibration bias and often resulting in a greater time depth for the estimate of viral origins^4^,^5^,^13^,^14^,^15^,^16^,^17^,^18^. However, the oldest specimens that have been used to date have been around 100–150 years in age^13^,^17^. It is therefore possible that further improvements on the estimate of age of virus origins could be obtained from viruses recovered from older, archaeological material.

The most studied plant virus that infects multiple crops is Barley Stripe Mosaic Virus (BSMV)^19^. BSMV is a tripartite, positive-strand RNA virus of the *Hordeivirus* genus, which has no vector and is transmitted through direct contact via pollen and seeds^20^. It is important both in terms of the economic damage it incurs in wheat and barley crops^21^, but also because it has become a tool for studying gene function in plants *in vivo* by virus-induced gene silencing methods. Furthermore, BSMV is capable of infecting hundreds of other members of the Poaceae including *Zea mays*, as well as some eudicots such as *Nicotinia* and *Chenopodium*^22^. The virus was discovered in 1950, and the earliest record of the symptoms associated with it was only 100 years ago^23^. Subsequently, BSMV has been found in North America, Europe and Asia and more recently in North Africa^24^,^25^. This disease therefore has the appearance of a recent origin and rapid spread. We were able to test this hypothesis by screening archaeological samples of barley spanning a range of ages from a few hundred to three thousand years for the presence of the virus, and used next generation sequencing to retrieve archaeogenomic levels of ancient RNA in order to attempt a genomic reconstruction.

## Results and Discussion

### Presence of BSMV in archaeological barley

The detection of ancient BSMV virus requires the preservation of ancient RNA in the archaeological record. To date there have been few studies on RNA^26^,^27^ because it is highly labile relative to DNA, with an expected 50-fold increased rate of degradation^28^. RNA is highly vulnerable to hydrolytic attack relative to DNA, but less prone to depurination^29^,^30^. Therefore in very dry environments, the rate of RNA degradation may be greatly reduced^30^. Archaeological barley samples were selected from Qasr Ibrim, a site in North Africa where BSMV has been reported. The arid conditions of the site are expected to be conducive to RNA preservation as has been observed for other biomolecules in samples of comparable age^31^,^32^,^33^. We tested grains of barley from Islamic, Late Christian, Meroitic and Napatan archaeological strata by RT-PCR of one target from each of the three genomic segments: the helicase gene (alpha segment; 123 nt), the beta-C gene (beta segment; 126 nt) and the RNA-dependent RNA polymerase gene (gamma segment; 137 nt), table S1. Amplicons were successfully produced only from the Late Christian sample (QI84/71) that is archaeologically dated to 600–900 years BP. This sample was subject to an archaeogenomic analysis of its small RNA fraction.

### Genome reconstruction and authentication of an archaeological BSMV virus

The small RNA fraction of sample QI84/71 was sequenced on the Illumina Hi-Seq platform. Allowing 1 mismatch per read of average length 20 nt, we assigned 49,232 non-redundant reads and 133,756 individual reads as BSMV. These were aligned to a reference genome in 51 contigs over 10,171 nucleotides with an average coverage depth of 300 reads and cross-genome coverage of 99.4%. From this data we constructed a consensus archaeological genome of the BSMV virus for further analysis. As a control we checked that the genome we had generated did not in any way reflect the Barley genome content. This may be expected from a retrovirus but was not expected from a *Hordeivirus*. *In silico* fragmentation (see methods) and Bowtie alignment of the archaeological virus genome to the barley (Morex) genome^34^ showed no continuous sequences of >50 nt present in the barley genome, even when allowing the maximum 3 mismatches under alignment parameters. We therefore conclude that the archaeological genome does not represent an unexpected form of barley genome incorporation of the virus.

While historical virus RNA of less than 100 years in age has been authenticated^35^,^36^,^37^, the conclusions of some previous studies of potentially older aRNA virus data have been attributed to contamination^38^,^39^. For further authentication we employed a method of comparative base modification profiles to establish the likelihood of this being a modern contaminant strain. In aDNA, one of the most common postmortem degradation markers is that of cytosine to uracil deamination by hydrolysis of cytosine by H_2_O^40^,^41^,^42^. We expected to observe a similar process in RNA as has been suggested in studies of RNA editing^43^. The proportion of C > U differences relative to other base change types of individual reads from the archaeological sample mapped to the BSMV genome contrasted significantly to that observed between published modern BSMV virus sequences and the consensus BSMV genome sequence, figure S1. We then compared the linear regression values of base changes between modern and archaeological strains, both with (R^2^ = 0.647) and without (R^2^ = 0.934) C > U transitions. We also performed an equality of variance F-test for both complete modification profiles (p = 0.12), showing that C > U transitions are more prevalent in the Qasr Ibrim strain than would be expected from *in vivo* processes alone. We confirmed likelihood of these transitions being the result of postmortem degradation by running BAM alignments of each segment against a reference strain using mapDamage v2.0^44^. Interestingly, the distribution of probable postmortem C > U transitions was more pronounced at 5′ and 3′ ends (figure S1c) where comparable DNA studies involve removal of 3′-overhangs as part of library preparation^44^. This could be due to the central portion of the single stranded RNA molecule being protected from hydrolytic attack through secondary structure leaving both ends overhanging and so more prone to deamination.

This evidence leads us to conclude that we have detected a genuine instance of BSMV from a ~750-year-old archaeological sample of barley, which is the oldest authenticated instance of an ancient virus, and the first archaeological virus genome that has been sequenced.

### The age of origin of BSMV

In order to reconstruct the evolutionary history of BSMV, we compared the archaeological genome sequence with six modern, complete genome sequences that have been published over the past 3 decades^45^,^46^,^47^,^48^,^49^,^50^. A previous study suggested a phylogenetic mosaicism between BSMV and the other two members of the *Hordeivirus* genus, poa semilatent virus (PSLV) and lychnis ringspot virus (LRSV), at different gene loci^20^. It would make the use of the whole genome in a phylogenetic analysis problematic if the different genes represented very different phylogenetic histories associated with separate species genomes. However, we found that PSLV consistently formed the closest sister taxon to BSMV at all seven genes that occurred in all three viruses, figure S2. We conclude that the evidence does not support a history of recombination between BSMV and LRSV. Therefore we selected PSLV as the outgroup in all our subsequent phylogenetic analyses.

**Figure 1:**
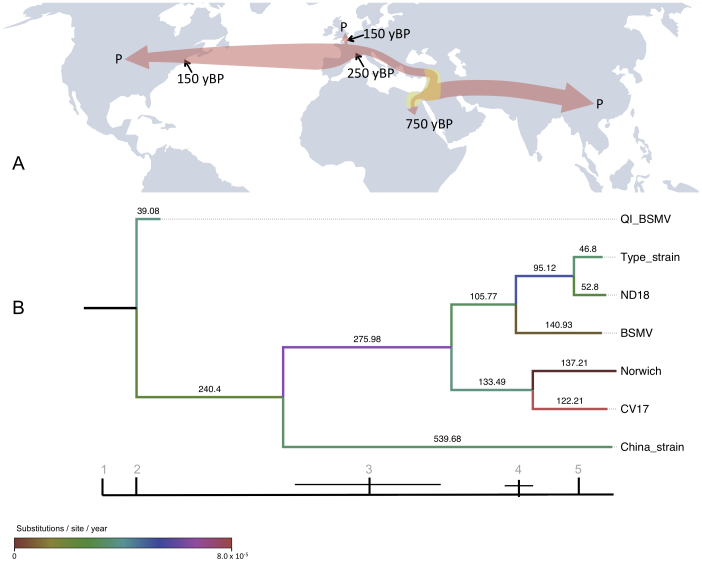
Hypothesised population movements and phylogeny of BSMV. (A): Global distribution map showing likely Fertile Crescent origin of BSMV ~100,000 yBP - present. The single yellow circle indicates probable area of origin of BSMV distribution prior to infection of Qasr Ibrim. Red arrows indicate probable transmission routes based on available strain data; West to East across Europe and to North America during the Industrial and Green Revolutions, and East to West along the Silk Road to China and East Asia during the medieval period. ‘P' indicated present day and applies globally as BSMV is thought now to be found worldwide. Map template provided by (http://www.freeworldmaps.net/pdf/maps.html). (B): BEAST analysis of all extant and Qasr Ibrim BSMV strains using an epidemiology model. The topology of BSMV using this model is consistent with that including an outlier (see Figure S3) with similar node dating and consistent clustering of western strains compared to archaeological or eastern strains. Relevant events indicated on timeline as follows. 1: MRCA of BSMV strains ~10,000 yBP (timeline unscaled). 2: Projected initial infection of BSMV at Qasr Ibrim, possibly terminated during the Late Christian era. 3: Re-opening of Silk Road trade route by Mongol Empire allowing the spread of BSMV into Asia. 4: Industrial revolution in Europe and the USA being a potential gateway from Europe to North America. 5: Postwar global Green Revolution; intensive farming providing greater opportunity for viral transmission and spread. Note variability in molecular evolution rates (branch colours denoted by key) with increased rates occurring at the root of western strains and more recently along the CV17 and Norwich strains.

We used BEAST to construct a heterochronous calibrated tree both in the presence and the absence of the archaeological BSMV sample, Figure 1 and Figure S3. A constant population size model was applied when comparing BSMV and PSLV as has been recommended for interspecific comparisons^51^. We applied an epidemiological model when analyzing diversity between different BSMV strains as has been recommended for intraspecific viral comparisons^52^, although we found little difference between these results and the application of a constant population model. We compared the marginal likelihood of both models by computing the Bayes factor through posterior distribution sampling. Since the harmonic mean of the sampled likelihoods is an estimator of the marginal likelihood, we calculated the harmonic mean of the likelihood from the posterior output of each model and extrapolated the difference in log space. We found the difference is too small to be significant so we did not reject the epidemiological model which also had the greater mean likelihood of the two models albeit marginally, Figure S5c.

We found that the inclusion of the archaeological BSMV virus calibration had a striking impact on the estimate of the age of the origin of the virus, a phenomenon reported previously given the inclusion of actual or virtual recalibration^37^,^53^. In the absence of the archaeological virus calibration, we estimate a mean substitution rate of 7.3 × 10^−4^ substitutions/site/year, figure S4. The base of the crown group dates to about 50 years ago, and the BSMV appears to split from PSLV about 150 years ago, figure S3. This data would seem to support the recent origin of the virus in keeping with its recent discovery and documented evidence of BSMV-like symptoms. However, our discovery of the virus in 750-year-old archaeological samples clearly challenges that scenario. The phylogenetic reconstruction using the archaeological virus places it close to the base of the crown group, suggesting that this sample was close to the origin of the spread of modern BSMV. The plesiomorphic position is supported by the lower number of substitutions on the branch leading to the archaeological virus, which further validates its authenticity, figure 1. In this reconstruction we find a range of substitution rates that are considerably lower than if the archaeological data are excluded (Figure S4). We find the common ancestor of BSMV and PSLV occurs within a large temporal range, the median date of the range correlates to the time of domesticated barley's appearance in the archaeological record^54^, Figure S5b. To explore the information value underlying the wide temporal range we plotted the frequency distribution of branch ages leading to PSLV from the origin, Figure S5d. We observe a strong modal signal at 2000 years BP, with 80% of the branches being less than 28000 years old. The age of the common ancestor is therefore likely to be in the younger part of the temporal range estimated from BEAST. It is possible that further instances of BSMV may be present earlier in the archaeological record and would alter the tree calibration if found as has been reported previously^37^.

### Evolution and spread of BSMV

The archaeological BSMV genome is close to the base of the crown of the phylogeny indicating that it is closely associated with the origin of modern populations of the virus. Interestingly, this dates to around to 1234 AD in the phylogenetic reconstruction, which corresponds closely to the seventh Crusade of Louix IX^55^. While such dating should be interpreted with caution because of the naturally associated wide margins of error (figure S6), it is interesting to note that this was one of the few Crusade episodes to visit North Africa, giving some credibility that this episode could have been instrumental in spreading the virus to Europe. The tree topology shows a clear divide between a single Chinese lineage, and the remaining lineages that are associated with Europe and America. This split in east and west lineages dates to around the 1473 AD, which is a century after the Mongol Empire stabilized the Silk Road. It is likely that BSMV was transported to the east via trade routes such as the Silk Road in the late Medieval period. While we can estimate that BSMV left for East Asia just over 500 years ago, in the absence of further East Asian BSMV genome sequences we cannot estimate the time that the virus arrived.

We observed variability in the substitution rate associated with the expansion of BSMV (Figure 1) a finding similar to that of a previous palaeoepidemiological study of the plague^56^. Notably, the initial branch leading to the European and American lineages shows an increased rate of 7.01 × 10^−5^ substitutions/site/year that may be associated with an early epidemic of the virus from the 15^th^–18^th^ centuries. A notable further rate increase was observed in the CV17 lineage in recent years. The Chinese lineage on the other hand shows no increase in rate that may indicate no particular epidemiological expansion in BSMV, perhaps reflecting the lower emphasis on barley in agriculture in the east. In more recent history, the virus appears to have spread to the US from Europe around 120–150 years ago whereupon an increased rate of substitution occurred, possibly caused by either a second epidemiological expansion or increased environmental or human-mediated selection pressure.

To investigate how the virus had changed functionally since the Late Christian period in North Africa we examined the non-synonymous substitutions that differentiated the archaeological BSMV from modern virus genomes. We identified 14 amino acid substitutions (table S2), none of which were obviously attributable to a change in function. Interestingly, most changes were located in the methyltransferase and polymerase coding genes, while only three occurred in other genes. Tertiary structure prediction of coding gene products using the SWISS-MODEL workspace showed no significant change in any identifiable domain when compared to the gene product of the BSMV reference strain^45^, where structure prediction was possible. Conserved amino acid positions in coding genes were identified by comparison with the wider gene family using pFam^57^. The archaeological sequence showed no differences in conserved positions to the BMSV reference strain. The archaeological genome therefore appears to represent a functional genome sequence, but it is unknown whether this virus would have had any difference in virulence to modern BSMV.

To survey for evidence of selection in the BSMV genome over the course of its history we examined the ratio of non-synonymous to synonymous SNPs (dN/dS) for each branch of the phylogenetic tree, Figure S6 and S7, and Table S2. The overall dN/dS ratio of the tree is high relative to the range expected for plant viral evolution^57^, which could be indicative of adaptive evolution across the BSMV tree. However, the proportions of nonsynonymous SNPs for Qasr Ibrim are low both compared to the other BMSV strains and the overall average expected for viruses. We conclude that it is unlikely that significant adaptive evolution occurred along this lineage. In contrast, the remaining strains show evidence of several episodes of adaptive evolution. The most significant dN/dS signal of adaptive change was found on the CV17 lineage, which was also associated with the greatest increase in the base substitution rate we observed in Figure 1. This is the only lineage to be highly significant relative to the other BSMV strains. However, all but two branches indicate a significant signal of selection when compared to expected plant viral range for dN/dS ratios. Generally, the strength of signal does not correlate closely with episodes of increased base substitution rates, rather a picture emerges of continual adaptive evolution over the history of the virus.

### An archaeogenomic perspective of BSMV origin

The calibration introduced by the inclusion of an archaeological virus profoundly alters the interpretation of the likely origin of BSMV. Our estimation of the BSMV rate of molecular evolution based on the archaeological data is approximately 3.9 × 10^−5^ substitutions per site per year, a slower rate than estimated by recent serial and heterochronous sampling. This study demonstrates that a virus that has the appearance of a recent origin, which could lead to policy decisions about movement of plant material, is shown to have a much older origin associated with historic or prehistoric activity. It is likely that early barley first contracted BSMV from a local wild grass population. Hordeiviruses rarely jump hosts because of a lack of vectors involved in their transmission. Consequently, direct contact between grains of the crop and grass weeds caught in cultivation is a likely mechanism of transmission and consequently origin in this case. It is possible that other viruses that similarly appear to be of very recent origin may in fact have a more ancient origin.

The inclusion of archaeological data allows the observation of genomic change over a relatively long time scale which includes periods of variable and adaptive evolution rates based on population movement, infection of new cultivars and changing environmental conditions according to those new hosts. These data point to a phylogeographic expansion of BSMV beginning around the time of the European Industrial Revolution and continuing into the 21^st^ century. Our data appear to indicate that until around 800 years ago, BMSV did not become widespread. We cannot estimate with any precision how long BMSV existed in the agricultural system before this expansion from these data. It may have crossed into barley during the initial domestication process, although a later entry a couple of thousand years ago into barley is more strongly supported by the data. It may be the case that BMSV did not become well established in agriculture until a threshold of intensity had been crossed in terms of density of barley stands and field sizes. In this case it could have been the demands on the local agricultural economy of the medieval war machines that required an extensive associated supporting population aside from the armies themselves that caused such a threshold to be crossed.

## Methods

### RNA extraction

RNA was extracted from archaeological barley grain, collected from Qasr Ibrim, an island/hill fort site in Upper Nubia (southern Egypt). Grain exhibiting BSMV infection came from the Late Christian strata only, dating from 600–900 years before present (catalogue number QI84/71). Ancient RNA was extracted in a dedicated clean lab facility meeting accepted criteria for authenticity of ancient biomolecules^58^. A modified protocol of the MirVana miRNA Isolation Kit (Ambion) was employed, with extended (8 day) initial incubation in 1 ml CTAB buffer (2% w/v CTAB, 1% w/v PVP, 0.1 M Tris pH 8.0, 20 mM EDTA, 1.4 M NaCl) at 37°C following grinding of seeds to powder with a mortar & pestle. RNA samples were extracted once with chloroform:isoamyl alcohol and precipitated with 1 volume isopropanol in 1.4 M NaCl prior to dissolution in guanidium buffer. RNA was isolated from DNA by a single extraction with phenol:chloroform:isoamyl alcohol at pH 4.2. RNA was then cleaned up with MirVana columns, according to the manufacturer's instructions. Final RNA extractions were quantified using the Qubit broad-range RNA assay (Invitrogen).

### Illumina library construction

Illumina libraries were built using a Small RNA Library Preparation Kit (Bioo Scientific) according to manufacturer's instructions. All pre-PCR steps of library construction from archaeological RNA took place in the dedicated clean facility. Sequencing was performed using the HiSeq 2500 platform (Illumina) using a single lane for the sample.

### Metagenomics

FastQ data was converted to FastA format and redundant sequences consolidated using scripts of the author's design, retaining frequency information within FastA tags. All sequences of abundance >1 were subject to BLASTn analysis using a custom 64-bit build of version 2.2.25 on a dedicated server. BLASTn output files were parsed for metagenomic data using MeGAn, where the presence of BSMV RNA was first identified in the archaeological dataset.

### Bowtie alignment

Detection of all potential BSMV reads from archaeological data was performed using Bowtie version 0.21. Bowtie indexes were constructed from the original BSMV reference strain (Genbank BioProject PRJNA15031) for each of three chromosomal segments. Alignment was performed using complete Illumina sequence data in consolidated FastA format, allowing for one mismatch per read and so constraining alignments to >95% accuracy.

### Control check against Morex genome

To check that the viral genome that was constructed was not an artifact from the host barley genome, the first was checked against the latter for regions of similarity. The viral genome sequence was randomly fragmented 3 times into 100, 50 and 18–21 nt sections (*in silico* fragmentation). An alignment was then attempted of each set of fragments against the Morex genome using Bowtie. No fragments of 50 nt or larger aligned, 129 fragments of 18–21 nt section aligned to the Morex genome accounting for 40 contiguous sequences of < 28 nt length and representing 9.7% of the total BSMV genome.

### Authentication by nucleotide deamination profiles

Archaeological reads positively aligned to the BSMV reference strain by Bowtie were parsed according to alignment specificity. To avoid statistical bias arising from high-frequency reads containing *in vivo* polymorphisms, base modification types of all non-redundant reads were collated from Bowtie output files and summarised, using a Unix shell script of the author's design. Further authentication of deamination was carried out with mapDamage 2.0 using standard parameters^44^.

### Genome assembly

Reads identified using these methods were aligned to the full BSMV genome in segments to visualize coverage depth using Geneious v5.1. 96.6% coverage was achieved across 51 intervals. The draft archaeological genome was aligned to 6 available BSMV reference strains, each comprising 3 segments (Genbank accessions NC_003469.1, NC_003481.1, NC_003478.1, J04342.1, X03854.1, M16576.1, AY789693.1, AY789694.1, AY787207.1, U35767.1, U35770.1, U13917.1, U35766.1, U35769.1, U13916.1, JF803283.1, JF803284.1, JF803285.1) using the Geneious align feature. For a second draft, sequence gaps were putatively filled first by re-alignment of short reads to the BSMV genome, allowing 3 mismatches as opposed to 1, giving 99.4% coverage. Where gaps existed after this alignment, the 62 remaining character states were inferred based on maximum likelihood character state reconstruction using Mesquite from the topology resulting from BEAST analysis of the first draft genome.

### Preliminary phylogenetic analysis

For nucleotide-based phylogenetic analysis, segments of the tripartite genome were concatenated into a single contiguous genome. Sequence gaps within the archaeological genome and corresponding bases in aligned genomes were removed so as to avoid bias in subsequent phylogenetic assignment. The closest relative within the *Hordeivirus* genus, Poa semilatent virus (PSLV) was chosen as an outlier and also subject to gap/nonaligned segment removal. Neighbor-joining trees were constructed based on this initial alignment to ascertain a guide phylogeny to preclude incorrect clade assignment as a result of using tip dates with BEAST.

### Phylogenetic analysis using Bayesian inference method

Phylogenetic placement based on available sequence data for 6 strains of BSMV and corresponding sections of PSLV as an outgroup was performed using BEAST using a relaxed lognormal clock and epidemiology tree prior. All BSMV strains were partitioned into a single monophyletic group for comparison to the outlier. Tip dates for each accession were defined by date of publication for extant strains and estimated as mid-period (Late Christian; 600–900 years BP) for the archaeological being 750 years old. A comparison Neighbor-joining tree was constructed with Geneious using the same alignment.

To preclude the possibility of given tip dates resulting in bias of branch lengths, manual calculation of relative branch lengths based on SNPs of the BSMV/PSLV alignment (3,351 bases) was performed by observing SNPs according to a branch model output by tip-dated BEAST analysis. The number of SNPs along each branch was used to calculate relative branch lengths.

### Calculation of molecular evolution rate

Molecular evolution rates of BEAST outputs from BSMV alignments only, and separate analysis including the PSLV outlier, were calculated using Tracer. Sequence gaps in the archaeological sample, their corresponding bases in extant strains and ambiguous bases in all strains were discarded so as not to bias analysis.

### Evaluation of potential selection pressures

Selective pressures identifiable though nonsynonymous SNPs (nsSNPs) were assessed across full genomes of 7 strains (6 full extant strains and the archaeological strain). Expected frequencies of nsSNPs for each strain (E) were calculated according to the probability of obtaining the observed number (O) of snSNPs based on the total length and the global frequency of non-synonymous SNPs. The Chi squared (χ^2^) statistic was used to calculate significance of deviation of observed and expected values.

## Author Contributions

O.S. carried out the research, R.G.A. designed the research project, A.C. and P.R. provided archaeological materials, Y.L. and J.W. provided sequencing facilities, O.S., R.G.A., A.C. and P.R. analyzed the data and O.S. and R.G.A. wrote the manuscript.

## Supplementary Material

Supplementary Information supplementary figures and tables
