# Supplementary material for: A complete ancient RNA genome: identification, reconstruction and evolutionary history of archaeological Barley Stripe Mosaic Virus
Source: Sci Rep. 2014 Feb 6;4:4003. doi: 10.1038/srep04003 (PMC3915304; doi:10.1038/srep04003)
Supplement: Supplementary Information — supplementary figures and tables [file srep04003-s1.pdf]

Supplementary Information

A complete ancient RNA genome: identification, reconstruction and evolutionary history of archaeological Barley Stripe Mosaic Virus

Oliver Smith[1], Alan Clapham[1], Pam Rose[2],Yuan Liu[3], Jun Wang[4], Robin G Allaby[1*].

1. School of Life Sciences, Gibbet Hill Campus, University of Warwick, Coventry CV4 7AL
2. The Austrian Archaeological Institute; Cairo Branch, Zamalek, Sharia Ismail Muhammed, Apt 62/72, Cairo, Egypt.
3. BGI-Europe-UK, 9 Devonshire Square, London, EC2M 4YF, UK.
4. BGI-Shenzhen, Shenzhen 518083, China.

* corresponding author email: r.g.allaby@warwick.ac.uk

Contents

Figure and Table legends……………………..3
Figure S1……………………………………………6
Figure S2……………………………………………7
Figure S3……………………………………………8
Figure S4……………………………………………9
Figure S5……………………………………………10
Figure S6……………………………………………11
Table S1……………………………………………...12
Table S2……………………………………………...13
Archaeological BMSV genome………………...14

Figure S1: Nucleotide substitution distributions. A: nonredundant small RNA reads of Qasr Ibrim BSMV to all extant strains after Bowtie alignment. C>U substitutions are highly prevalent in ancient DNA as a result of diagenesis, and similar profiles can be expected from ancient RNA. False identification of *in vivo* SNPs resulting from overrepresentation of postmortem C>U substitutions is unlikely due to average coverage depth of 300 after Illumina sequencing. B: Mean phylogenetic polymorphism counts based on alignment of Qasr Ibrim genome to all extant (full) genomes. C: Posterior prediction of postmortem C>U transitions following short RNA read analysis with mapDamage 2.0.

Figure S2: BEAST analysis of individual genes identified from all available *Hordeivirus* sequence data. Panel A: methyltransferase gene. Panel B: coat protein gene. C: Beta-D gene. D: putative helicase gene. E: transport protein gene. F: Gamma-B gene. E: RNA-dependent RNA polymerase gene. All genes show greater homology of PSLV to BSMV than of LRV to BSMV.

Figure S3: BEAST tree based on constant population size showing calibration without the presence of archaeological BSMV. Note the significant (~tenfold) reduction in estimated basal node age.

Figure S4: TRACER nucleotide substitution rate estimates from BSMV genome sequence data. A: The mean substitution rate in the presence of archaeological sequence data is considerably lower than examining extant strains only (B). This suggests that BSMV emerged earlier than previously thought and is consistent with

the hypothesis of co- or near co-emergence of pathogen to host during plant domestication.

Figure S5: BEAST analyses showing median node ages and 95% HPD range (blue bars). A: epidemiology model of BSMV strains. B: constant population model of BSMV and PSLV strains. HPD range of node 1 (11248.3864 median) bar has been adjusted; actual range shown in red lettering. Refer to table S1 for ranges. C: comparison of marginal likelihoods of constant population and epidemiological models following Bayesian inference of BSMV strains using both models. D: Frequency distribution of branch age estimates to MRCA of BMSV and PSLV, median shown by red bar.

Figure S6: A: Branches labeled 1-11 for reference to table S2. B: Frequency distribution of dN/dS ratios after Murray (et al., 2013). The dotted line represents the BSMV dN/dS ratio taken from SNPs of all strains.

Table Legends

Table S1: 95% highest posterior density (HPD) interval ranges and median node ages for BEAST models.

Table S2: dN/dS breakdown. Branches from figure S6 and associated synonymous SNPs, non-synonymous SNP to give dN/dS ratios according to established tree topology. Ratios calculated manually according to synonymous and non-synonymous SNPs unique to each branch. Chi-squared probabilities of branches from established tree topology (see figure S6). E_1: expected number of non-synonymous SNPs based

on observed dN/dS ratios. T_1: Chi-squared test statistic based on observed BSMV ratios in this study. P_1: Probability value associated with E_1 and T_1. E_2: expected number of non-synonymous SNPs based of mean dN/dS ratio of plant viral strains after Murray (et al., 2013). T_2: Chi-squared test statistic based on hypothesized dN/dS ratio. P_2: probability value associated with E_2 and T_2.

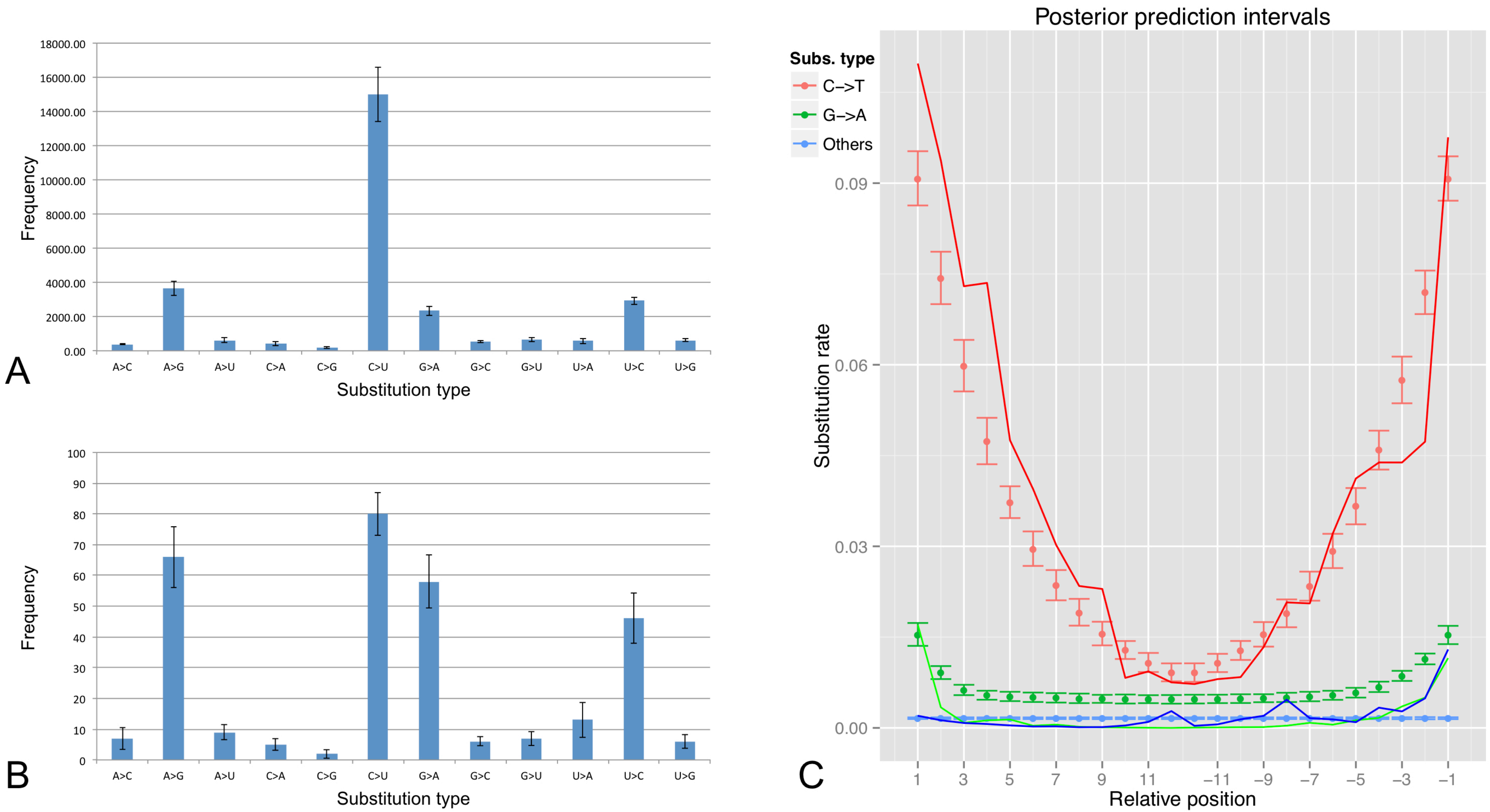

Frequency
Substitution type
A>C
A>G
A>U
C>A
C>G
C>U
G>A
G>C
G>U
U>A
U>C
U>G
A
B
Posterior prediction intervals
Subs. type
C−>T
G−>A
Others
Substitution rate
Relative position
C

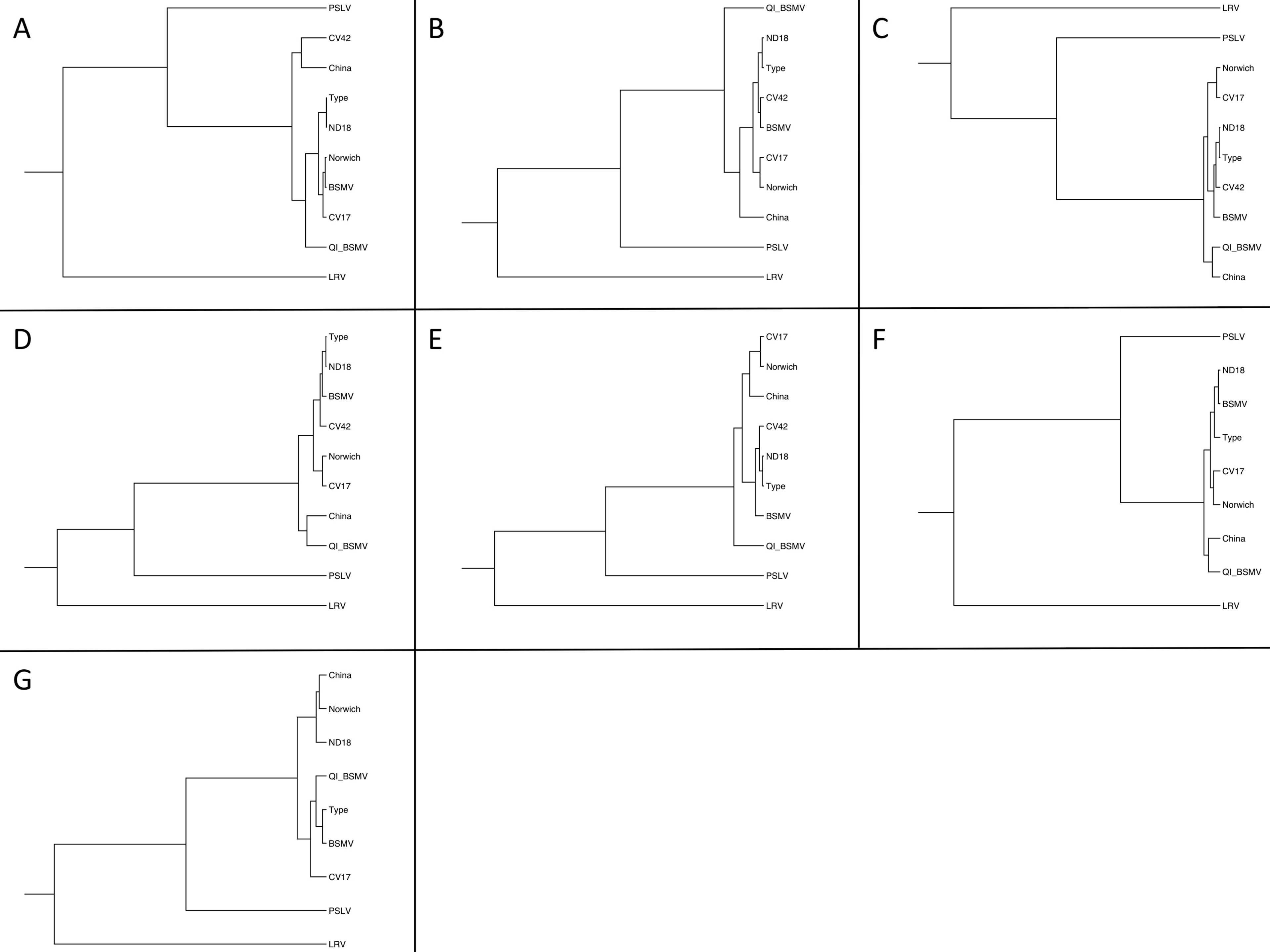

A
PSLV
CV42
China
Type
ND18
Norwich
BSMV
CV17
QI_BSMV
LRV
B
QI_BSMV
ND18
Type
CV42
BSMV
CV17
Norwich
China
PSLV
LRV
C
LRV
PSLV
Norwich
CV17
ND18
Type
CV42
BSMV
QI_BSMV
China
D
Type
ND18
BSMV
CV42
Norwich
CV17
China
QI_BSMV
PSLV
LRV
E
CV17
Norwich
China
CV42
ND18
Type
BSMV
QI_BSMV
PSLV
LRV
F
PSLV
ND18
BSMV
Type
CV17
Norwich
China
QI_BSMV
LRV
G
China
Norwich
ND18
QI_BSMV
Type
BSMV
CV17
PSLV
LRV

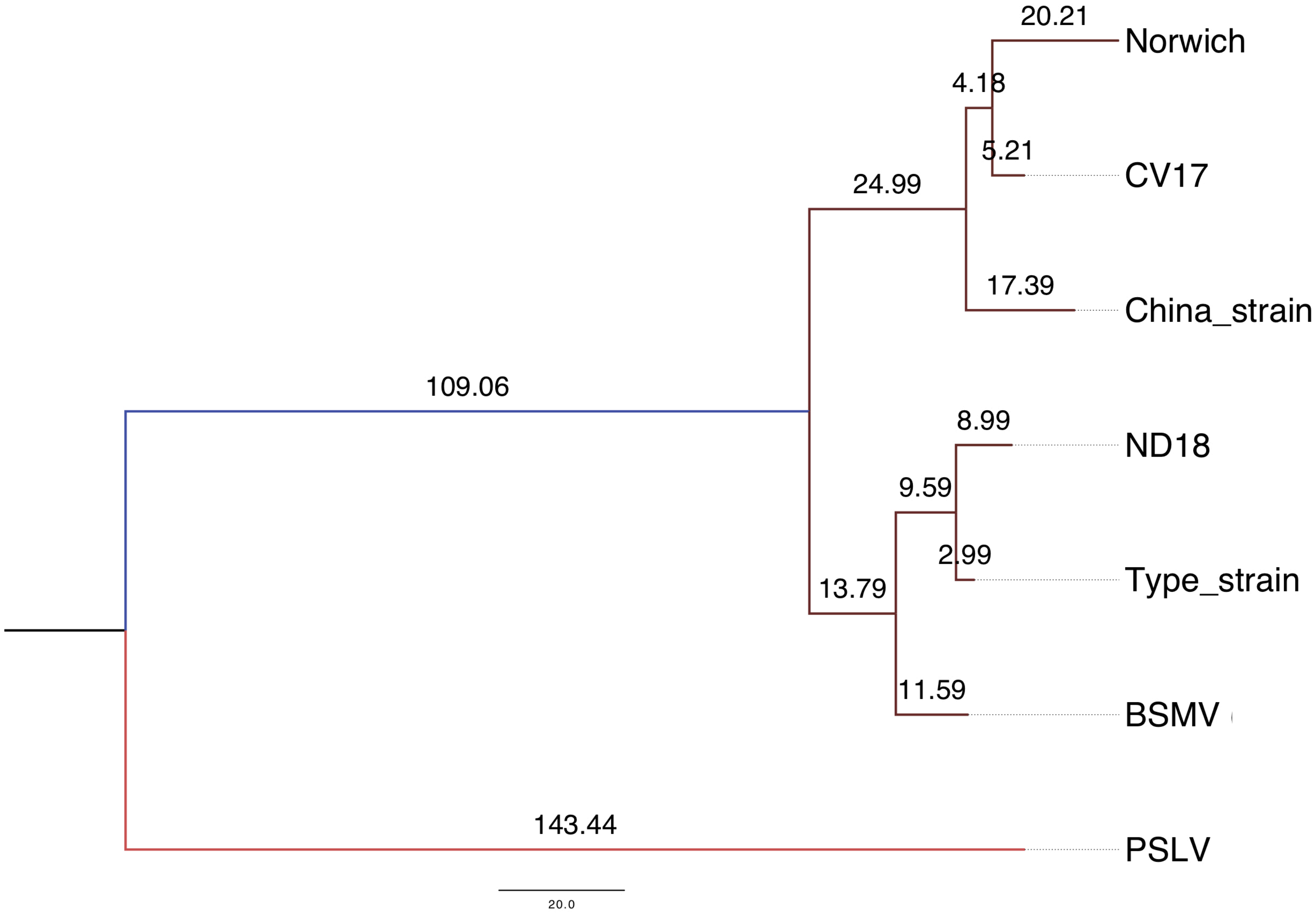
20.21
Norwich
4.18
5.21
CV17
24.99
17.39
China_strain
109.06
8.99
ND18
9.59
2.99
Type_strain
13.79
11.59
BSMV
143.44
PSLV
20.0

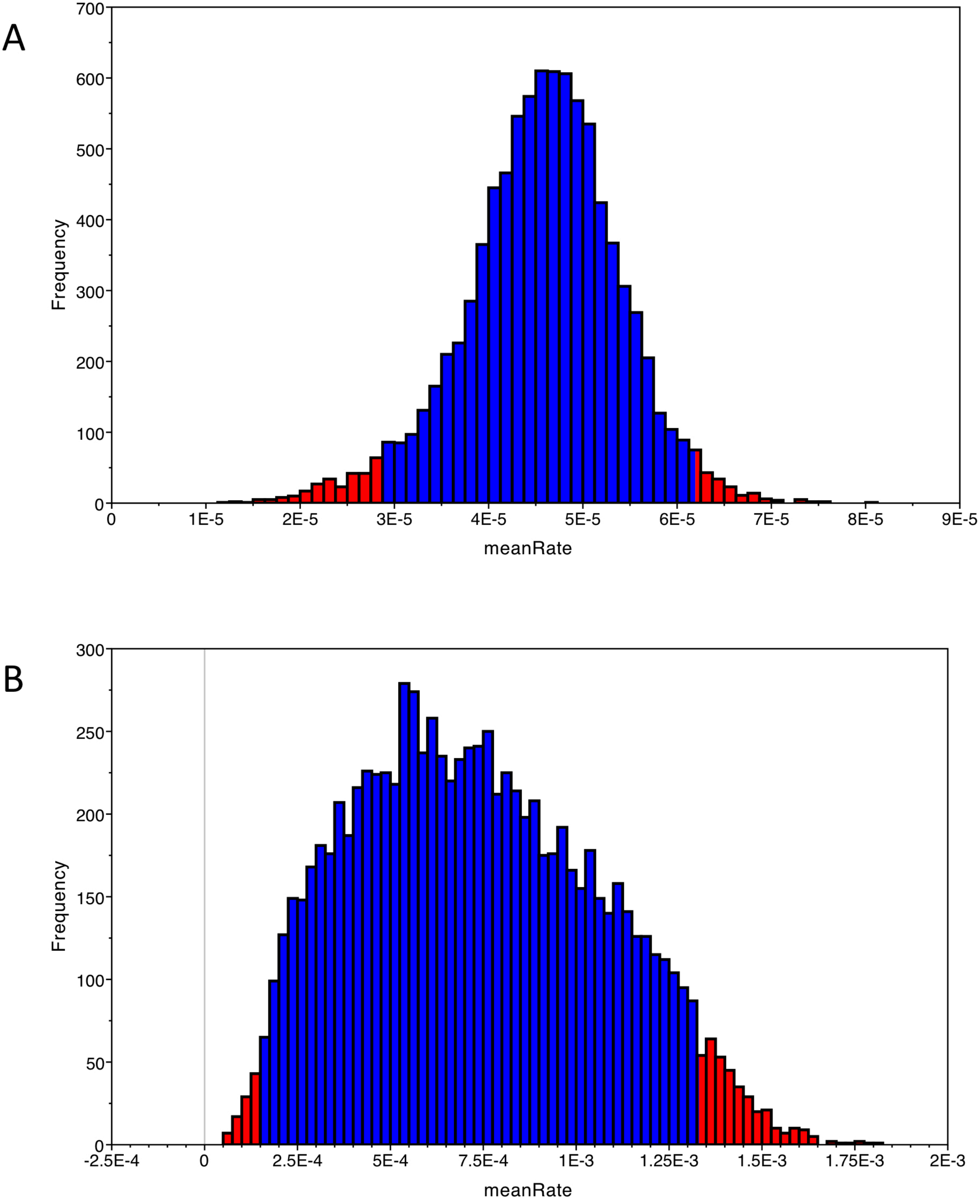
A
700
600
500
400
300
200
100
0
Frequency
0
1E-5
2E-5
3E-5
4E-5
5E-5
6E-5
7E-5
8E-5
9E-5
meanRate
B
300
250
200
150
100
50
0
Frequency
-2.5E-4
0
2.5E-4
5E-4
7.5E-4
1E-3
1.25E-3
1.5E-3
1.75E-3
2E-3
meanRate

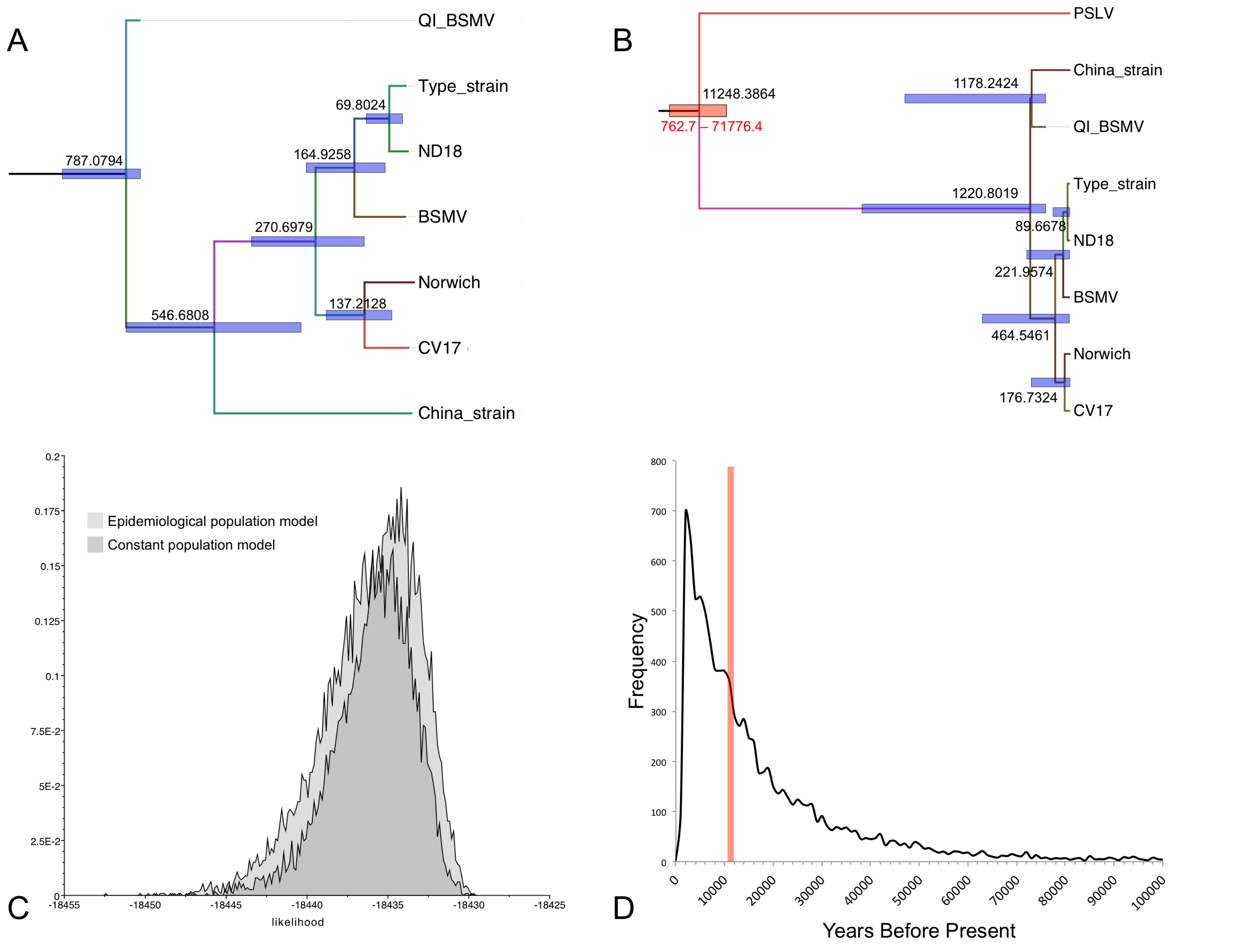
A
QI_BSMV
Type_strain
69.8024
ND18
164.9258
787.0794
BSMV
270.6979
Norwich
137.2128
546.6808
CV17
China_strain
B
PSLV
China_strain
1178.2424
11248.3864
762.7 – 71776.4
QI_BSMV
Type_strain
1220.8019
89.6678
ND18
221.9574
BSMV
464.5461
Norwich
176.7324
CV17
C
Epidemiological population model
Constant population model
likelihood
D
Frequency
Years Before Present

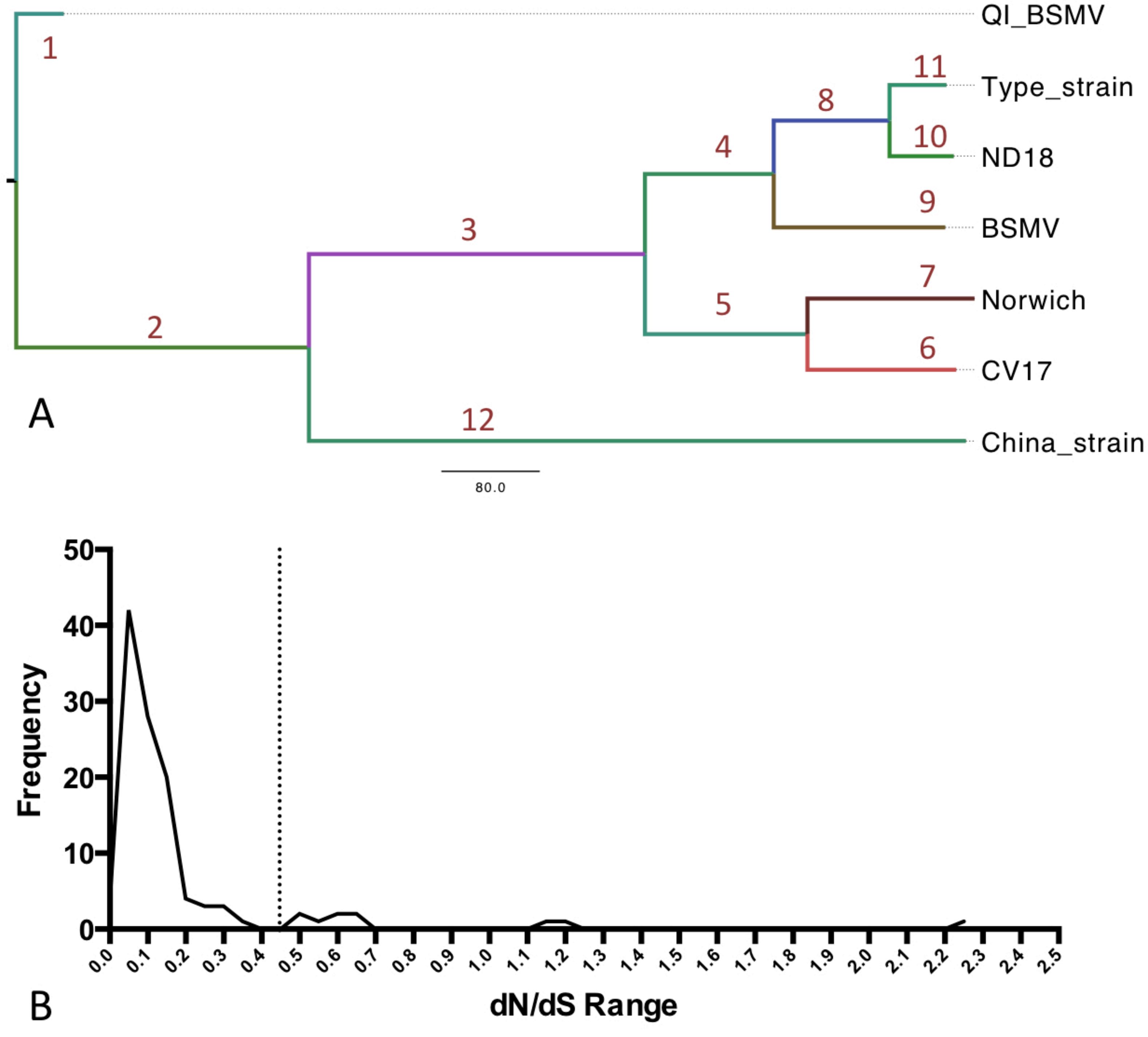

QI_BSMV
Type_strain
ND18
BSMV
Norwich
CV17
China_strain
1
2
3
4
5
6
7
8
9
10
11
12
A
80.0
Frequency
dN/dS Range
B
0
10
20
30
40
50
0.0
0.1
0.2
0.3
0.4
0.5
0.6
0.7
0.8
0.9
1.0
1.1
1.2
1.3
1.4
1.5
1.6
1.7
1.8
1.9
2.0
2.1
2.2
2.3
2.4
2.5

Table S1: 95% highest posterior density (HPD) interval ranges and median node ages for BEAST models.

**Model**	**Node age (median)**	**Range (HPD 95%)**
Constant with PSLV	11248.3864	762.6955 – 71776.3877
	1220.8016	748.0299 – 6318.3919
	1178.2424	755.7080 – 5018.1534
	464.5461	36.4178 – 2674.6957
	221.9574	27.6898 – 1323.1335
	176.7324	16.2921 – 1185.1729
	89.6678	23.6281 – 530.5303
Epidemiology with BSMV only	787.0794	748.0003 – 960.8303
	270.6979	138.1570 – 445.0934
	546.6808	310.1860 – 786.7864
	137.2128	62.8038 – 241.4581
	164.9258	81.0792 – 295.9000
	69.8024	33.6436 – 132.1580

Table S2: dN/dS breakdown. Branches from figure S6 and associated synonymous SNPs, non-synonymous SNP to give dN/dS ratios according to established tree topology. Ratios calculated manually according to synonymous and non-synonymous SNPs unique to each branch. Chi-squared probabilities of branches from established tree topology (see figure S6). E_1: expected number of non-synonymous SNPs based on observed dN/dS ratios. T_1: Chi-squared test statistic based on observed BSMV ratios in this study. P_1: Probability value associated with E_1 and T_1. E_2: expected number of non-synonymous SNPs based of mean dN/dS ratio of plant viral strains after Murray (et al., 2013). T_2: Chi-squared test statistic based on hypothesized dN/dS ratio. P_2: probability value associated with E_2 and T_2.

**Branch**	**Syn. SNP**	**ns SNP**	**dN/dS**	**E_1**	**T_1**	**P_1**	**E_2**	**T_2**	**P_2**
1	30	6	0.2000	13.42	4.10	0.0429	4.93	0.23	0.6303
2	64	26	0.4063	28.62	0.24	0.6239	10.52	22.78	0.0000
3	46	15	0.3261	20.57	1.51	0.2192	7.56	7.32	0.0068
4	10	6	0.6000	4.47	0.52	0.4701	1.64	11.55	0.0007
5	13	6	0.4615	5.81	0.01	0.9385	2.14	6.98	0.0082
6	8	14	1.7500	3.58	30.36	0.0000	1.31	122.37	0.0000
7	7	3	0.4286	3.13	0.01	0.9411	1.15	2.97	0.0847
8	9	5	0.5556	4.03	0.24	0.6270	1.48	8.38	0.0038
9	3	3	1.0000	1.34	2.05	0.1522	0.49	12.74	0.0004
10	6	1	0.1667	2.68	1.06	0.3041	0.99	0.00	0.9889
11	3	4	1.3333	1.34	5.27	0.0217	0.49	24.94	0.0000

Supplementary information: BMSV Genome Sequence
>QI_BSMV_alpha
GTATGTAAGTTGCCTTTGGGTGTGAAATTTCTTGCATGCACATAATCGTAATCGATTCaTCGTGATCTCTAAACA
ACACTTTCCCGTTAGCATGGCTAGCGATGAGATTGTCCGTAATCTGATCTCCCGTGAGGAGGTGATGGGTAATTT
GATTAGCACAGCTTCCAGTTCAGTAAGGTCGCCCTTACATGACGTACTGTGCTCGCACGTAAGGACCATCGTCGA
TTCCGTGGATAAGAAAGCGGTCAGTCGCAAGCATGTtGATGTACGGCGCAACATCTCCTCTGAGGAGTTGCAGAT
GTTGATAAATGCATATCCTGAATATGCCGTTTCATCTTCAGCTTGTGAATCTGGTACTCATAGCATGGCGGCTTG
TTTTCGATTTCTGGAGACAGAATATCTTTTAGATATGGTTCCAATGAAAGATACTTTTGTctatgatattggggg
tAACTGGTTTTCTCACATGAAGTTTCGTGCTGACAGAGAAATTCATTGTTGCTGTCCAATCTTATCTATGAGAGA
TTCTGAAAGACTGGAAACACGCATGATGGCAATGCAAAAATATATGCGTGGATCGAAAGACAAACCGTTACGCTT
GCTAAGCCGTTATCAAAATATCCTGCGTGAACAAGCGGCGAGAGCAACTGCCTTTgtgGCAGGTGAGGTGAAcGC
GGGTGTTCTCGATGGAGAGGTGTTTTGTGAGAACACTTTTCAAGACTGTGTGAGAGaGGTGCCCGAAGGTTTTTT
GAAGACAGCTATAGCAGTTCATAGCATTTACGATATCAAAGTGGAAGAATTTGCGTCTGCATTGAAAAGAAAAGG
TATAACACAGGCTTATGGGTGCTTCCTGTTTCCTCCTGCtGTATTGATAGGTCAGAAGGAAGGTATTTTACCTTC
CGTGGACGGTCATTATTTGGTGGAGAATGGCAGGATTAAGTTTTTCTTTGCAAATGATCCGAATGCCGGTTACTC
GCACGACCTTAAGGATTATCTGAAGTATGTGGAAAAGGCCTATGTGGATATAAAGGATGGAGTGTTTGCTATTGA
GCTGATGCAAATGCGAGGTGATACCATGTTCTTTAAGATCACGGACGTCACTGCAGCAATGTATCATATGAAATA
CAGAGGAATGAAACgtGACGAAACATTCAAATGCATTCCGATGCTAAAGAATTCATCTGTTGTCGTACCTCTATT
TTCATGGGACAatcATTCTTTAAAGATCACAAGTGGTTTACTACCACGAACTTTGGTCGAGCAGGGTGCGGCATT
TATTatgaaaaacaaggaaAAAGACTTGAACGTTGCTGTGTTGAAGAATTATCTTTCCGCTGTGAACAATTCATA
CATTTTCAACGGATCTCAGGTTAGAGATGGTGTGAAAATTGCTCCGGATTTAATCTCCAAATTGGCAGTGACTCT
GTTCCTGAGAGAAAAGGTCTATCGACAAAGAGAAAATTCAATCATAAGTCATTTCGAACAAGAAATGCTTCGCGA
TCCCAACTTGAAAGCCATGTTTGGAGACTTTCTGTGGTTTGTTCCAAATACTCTTTCGAGTGTTTGGAAGAACAT
GCGAAAATCACTGATGGAATGGTTTGGTTACGCAGATTTTGACTTGACTACTTTTGATATTTGCGATCCCGTTCT
CTATGTAGAGATAGTGGATCGGTATAAGATCATTCAAAAAGGGCGAATTCCACTTGGTGAGTTCTTTGATTGTCA
TGAAGAATGCGAGAATTACGAACTACGTGAGAAGGAGAAAAATGACCTAGCGGTGAAAATGGCCCAGAAGGTAAC
AGGGACGGTGACCGAATGCGAGAAGGACGagggacatcttgttcagccgacaaaacagatactagttcaacttat
gatgccccatattGTCAGAGCGCTGTGTAAACCTCGTAGCCCAACGTCTCCTTTGGACTTtagcagTATCCCAGG
GTCAACTCCATCACACTCAAGTTCAGATTCTGAACAATCTATGACTGAAGAAGCGAGCTTatcCATTGCGGGTAG
CGTACCAACATGGGAAATTGCGACTAAGAAAGATCTAACCTTTCAGCGAATTAATGAAGATATGTCTCGACGAAC
TGGTATGCCTCCAAGACCAAAAGTAACTTCCAGTTACAACATGAACGCCAGAGCCGAGTTTCTCTACTATCAACT
GTGTAGCGTGATTTGTGAAAGGGCCCAGATTTTGAGTGTCATCGAAGACTTTCGTCaaaactTGATATTCTCAGA
TAAGGTGGCCGTTCCATTGAACGCTAGATTTTACAGTTTTCAGTCgttgYaacctggatgggtgtacaagACTCC
ATCGTATAGTGAAGTAGGCCACAGTTATGCAGTACATTTTGACTTCAAGACGATTggaaatgagTTGGAAGAAAG
CCTAGCTTTTTGCCGATTGGTACCGATTTCATGGGATAAAAGCGGCAGATACATCGCGACAACTCCTTATTTTCC
CGAGAGACATGGTTACTACGTGATATGTGACAACACTAAATTGTGTAACAATTGGCTTATTTACAATAAGTTAGT
TGACGTCTACGCATTgGTGACTGATAGACCTCTGAAATTCGAGTTGATTGACGGAGTTCCTGGCTGTGGAAAGTC
tacTATGATTTTAAACAGCTGTGATATTCGACGCGAAGTTGTTGTTGGTGAAGGACGGAATGCGACTGATGACTT
AAGGGAGAGGTTCAAGCGAAAGAAAAATTTGAatagtaaaatTGCTAATCATAGAGTTCGAACGCTTGACAGCTT
ATTACTTGCTGAAGGACCTTGTATACCGCAAGCTGATAGGTTTCATTTTGATGAAGCTCTAAAAGTTCATTACGG
TGCTATAATGTTCTGTGCTGATAAGCTTGGTGCCTCAGAAATTCTCGCTCAAGGAGATAGGGCTCAATTGCCAAT
GATCTGTCGTGTAGAAGGAATTGAACTTCAATTTCAGTCTCCTGATTACGCGAAGACGATCATAAATCCTAAGCT
ACGATCATACCGTATCCCTGGGGATGTTGCCTTCTATTTGAGTGCTAAGGAGTTTTACAAAGTTAAAGGAGTACC
cTAAAAGGTTACAACTTCTAACAGTGTGAAGCGTTCCTTGTACGCTAGAGGTGAAACaACTCAGGAAAGATTCGT
GAGTTTGCTTGATGTTCCAGTGAGAAAAGACACCCACTATCTAACCTTCTTACAAGCTGAGAAGGAAAGTTTGAT
GAGTCATTTGATTCCAAAGGGTGTGAAGAAAGAGTCTATTTCAACGATTCATGAGGCACAGGGTGGTACCTATGA
AAATGTGATTCTGGTCCGTTTGCAACGGACTCCCAACGAAATTTATCCGGGTGGACTTAGGTCCGCCCCTTACAT
CGTGGTTGGGACTTCAAGGCATACGAAAACTTTCACTTATTGTAGTGTTACGGACGATAAGTTGCTTTTAGATAT
CGCCGATGTCGGTGGTATTGCACATACACCTATTCGTACTTTTGAGTCTCATATAGTttgAAAAAAAAAAAAAAA
TGTTTGATCAGATCATTCAAATCTGATGGTGCCCATCAACCATATGATGGGAGTGTTTACAAGTCCACTATAATC

GAACTTGAAAACCTTGCCTGAATTGGAAACCATGAATCTTAACGGATTCTGGAGAGAAAATTTAGGAATTGGTAT
GTAAGCTACAACTTCCGGTAGCTGCGTCACACTTTAAGAGTGTGCATACTGAGCCGAAGCTCAGCTTCGGTCCCC
CAAGGGAAGACCA
>QI_BSMV_beta
*gtaaagaaaac*GGAGCAACCCTGTTGTTGTTCGACACTA*aact*AAATATATATTATCTTATTAGTGCATTTCTT
TTACCGCTTCACAGTATGCCGAACGTTTCTTTGACTGTTAAGGGTGGAGGACACTATAACGAGGATCAATGGGAT
ACACAAGTTGTGGAAGCCGGAGTGTTTGACGATTGGTGGGTTCACGTAGAAGCCTGGAATAAATTTCTAGACAAT
TTACGTGGTATTAACTTTAGCGTTGCTTCCTCTCGGTCGCAAGTTGCTGACTACTTAGCTGCGTTAGATCGTGAT
CTACCTGCTGATGTAGACAGAAGGT*ttgctggtgctagaggaacaatc*GGTTTACCCAATTATCTTCCTGCACCA
AAATTCTTTCGTCTCGATAAGCGAACTATTGCTGAACTGACTAGACTCTCTCGTCTTACGGATCAGCCGCACAAC
AATCGCGATATAGAGCTTAACCGAACGAAAAGAGCCACAACTAACCCATCTCCCCCGGCGCAGGCACTG*ttggag*
*actcc*TACTCTTCGTGATGTTCAACCGTTGAAGGATAGTGCGTTGCATTATCAATACGTGTTGATTGACCTACAG
AGTGCTAGACTCCCAGTGTATACCAGGAAGACTTTCGAACGTGAACTCGCTTTGGAATGGATCATTCCAGATGCC
GAGGATGCGTGACCTGCTGTTGAAGCGGTAAAAGGATGTACATATGTATCTTAATTATTTTGTTTAT*t*TATTTTC
TTTTACTTTTAGTTTTTGCTTTTTACGCTTTAACTAGATGTATTGACTTTAGCCATGGACATGACGAAAACTGTT
GAGGAAAAGAAAACAAATGGAACTGATTCAGTGAAAGATGTTTTTGAAAACTCGACGATTCCCAAAGTTTCGACT
GGAAAGGAAATGGGTGGTGACGATTCTTCTACTTCTGAATTAAGGGAAACTCTGAAAGTTGCCAATCAGACTCCA
TTGTCCGTTGATAATGGTGCCAAATCCAAATTGGATTCTTCT*ggtagacaagttcctag*AGTTGCCAATCAGACT
CCATTGTCCGTTGATAATGGTGCCAAATCCAAATTGGATTCTTCT*ggtagacaagttcctaatcctgagttgaat*
*cacaaaa*TTAAGAAGTCCAAGAAGAAAAGAAACCAAAAACCTGCTCAACCGAGTAGGCCCAATGACCTTAAAGG*a*
*gagg*CTAAGGGATCATCTCAAGTGGGTGAGAATGTGAGTGAGAACTATACTGGGGTTTCTAAGGAAGCAGCTAAG
CAAAAGCGGAAGACGCCTAAGTCTGTGAAAATGCAAAGCAATCTGGCCGATAAGTTCAAAGCGAATGACACTCGT
AGATCAGAATTAATTAACAAGTTTCAGCAGTTTGTGCATGA*ga*CCTGTCTTAAGTCTGATTTTGAGTACACTGGT
CGACAGTATTTCAGAGCTAGATCAAATTTCTTTGAAATGATTAGGCTCGCATCCTTGTATGACAAACATCTAAAG
GAGTGTATGGCGCGAGCCTGCACCCTAGAACGAGAACGATTGAAGCGTAAGTTACTCTTAGTACGGGCTTTGAAA
CCAGCAGTGGANTTTCTTACGGGAATCATCTCTGGAGTTCCTGGCTCAGGAAAATCAACCATTGTGCGAACTTTG
CTCAAAGGTGAATTTCCTGCTGTTTGTGCTTTAGCCAATCCTGCCTTAATGAACGACTATTCTGGTATTGAAGGC
GTTTACGGGTTAGATGACCTGTTGCTTTCTGCAGTTCCGATAACGTCTGATTTATTGATCATAGATGAATATACA
CTTGCTGAGAGCGCGGAAATCCTGTTGTTACAACGAAGACTCAGAGCCTCTATGGTGTTGTTAGTCGGGGATGTA
GCTCAAGGAAAAGCCACCACTGCTTCTAGTATTGAGTATTTAACTCTGCCGGTGATCTACAGATCAGAGACGACT
TATCGTTTGGGACAAGAGACTGCTTCGCTTTGCAGCAAGCAGGGTAACAGAATGGTTTCAAAGGGTGGAAAGGAC
AAAGTGATCATTACTGATTACGATGGCGAAACAGATGAAACGGAGAAAAATATCGCTTTTACTGTCGATACAGTT
CGAGATGTGAAAGATTGTGGGTACGATTGTGCCCTGGCAATTGATGTGCAAGGGAAAGAATTCGATTCAGTGACT
TTATTCCTAAGGAACGAAGACCGGAAAGCTTTAGCAGATAAGCATTTGCGTTTAGTCGCTTTGAGCAGACATAAG
TCGAAGTTAATCATCAGGGCCGACGCGGAAATTCGTCAAGCATTCCTGACAGGTGATATTGACTTGAGCTCTAAG
GCGAGTAACTCTCATCGTTATTCTGCAAAACCGGATGAAGACCACAGTTGGTTCAAGGCCAAATAAGTATTGGCC
AATTGTCGCCGGAATCGGTGTCGTTGGATTGTTTGCGTATTTGATTTTTTCAA*atc*AAAAACATTCCACGGAATC
CGGTGATAATATTCACAAATTCGCTAACGGAGGTAGTTACAGAGACGGGTCAAAAAGTATAAGTTATAATCGCAA
TCATCCTTTTGCCTATGGCAATGCCTCATCCCCTGGAATGTTGTTGCCCGCAATGTTTACCATCATCGGAATCAT
TTCCTACTTATGGCGAACAAGAGATTCCGTGCTCGGAAACTCAGGCGGAAACAACTCCTGTGGAGAGGACTGTCA
GGGCGAGTGTCTTAACGGACATTCTCGACGATCATTACTATGCGATATTGGCTAGTCTTTTTATCATTGCTCTAT
GGCTATTGTATATATATCTAAGCAGTATACCTACGGAGACTGGTCCTTACTTCTATCAAGATCTAAACTCTGTGA
AGATCTATGGAATAGGGGCTACGAATCCGGAAGTTATTGCGGCCATCCATCATTGGCAGAAATACCCTTTTGGGG
AATCTCCGATGTGGGGAGGTTTAGTCAGTGTTTTAAGTATTCTACTTAAACCGCTGACGTTAGTTTTAGCGTTAA
GCTTTTTTCTCTTGCTTTCTTCAAAAAGGTAAAA*aa*AAAAAAAAAAAAAAATGTTTGATCAGATCATTCAAATCT
GATGGTGCCCATCAACCATATGATGGGAGTGTTTACAAGTCCACTATAATCGAACTTGAAAACTTTGCCTGAATT
GGAAACCATGAATCTTAACGGATTCTGGAGAGAAAATTTAGGAATTGGTATGTAAGCTACAACTTCCGGTAGCTG
CGTCACACTTTAAGAGTGTGCATACTGAGCCGAAGCTCAGCTTCGGTCCCCCAAGGGAAGACCA
>QI_BSMV_gamma
GTATAGCTTGAGCATTACCGTCGTGTAATTGCAACACTTGGCTTACCAAATAACGCTAGAGCGTTCACGAAACAA

AtcaaaacTTTGGCATGGATGTTGTGAAGAAATTCGCCGTCATGTCAGTGACTGTAGTAGCAGGTCCCGTCCTTA
CGTTctcCTCACCTGTGGTGGTGACGTTTGGAACAGGCTTAATTGCCGTATCTTTGGTGAAACGGTTGTTACAGG
AACACCCCCGTGTACTTGCTCACGATCACGAACATTGCCCAGGTGGTTCTGAGAGCAGTTCTAGCTCTTGTGCTA
CCGCGCCTATTCTACGTAATCTTTtgCAAGATCAGTGCGACTCAGAGAATATTGGATGCAATTCTAGCGCCTGTT
CTCCGTCTGGAATTGTGAAAGTTACAAGGCAGGTGGTGGAAGTTGAGCGTGGTCTTTACCGGGAcAtaaaatTTT
GGCATGGATGTTGTGAAGAAATTCGCCGTCATGTCAGTGACTGTAGTAGCAGGTCCCGTCCTTACGTTgttTTCA
CCTGTGGTGGTGACGTTTGGAACAGGCTTAATTGCCGTATCTTTGGTGAAACGGTTGTTACAGGAACAcccCCGT
GTACTTGCTCACGATCACGAACATTGCCCAGGTGGTTCTGAGAGCAGTTCTAGCTCTTGTGCTACCGCGCCTATT
CTACGTAATCTTTtgCAAGATCAGTGCGACTCAGAGAATATTGGATGCAATTCTAGCGCCTGTTCTCCGTCTGGA
ATTGTGAAAGTTACAAGGCAGGTGGTGGAAGTTGAGCGTGGTCTTTACCGGGATATTTTTCAGGACAACGAAATT
CCATCAGTCATGGAAGAGAAACTCCAGAAACTgctttattcaGAGGGTGAGAAAATTCGAAGACGTTGTCAATTT
GAAGCATCAACGATGCACTCACGCAAAGTAAAGGTTCCGGAGGTAGGTACTATCCCAGATATCCAAACTTGGTTC
GATGCTACGTTTCCTGGTAATTCCGTTAGGTTTTCTGACTTCGACGGTTATACTGTTGCTACGGAGGACATTAAC
ATGGATGTTCAGGATTGTAGACTTAAGTTCGGGAAGAATTTTCGACCGTATGAATTTAAGGAATCACTGAAACCA
GCACTGAGGACAGCAATGCCAGAAAAGCGACAGGGTAGTTTGATTGAAAGTGTGCTGGCCTTTCGTAAAAGAAAT
TTGGCTGCGCCCAGATTGCAgGGAGCTTTGAATGAATGGCATACAATTGAGAATGTGCTGAAGAAGGCGTTAAAG
GTATTCTTCTTTGAAGATTTAATTGATCGGACGGATCATTGCACTTACGAGTCAGCACTCAGATGGTGGGATAAA
CAATCAGTGACAGCTCGAGCACAGCTCGTGGCGGACCAACGGAGGTTATGTGATGTTGACTTCACGACTTATAAC
TTCATGATAAAAAATGATGTAAAGCCGAAGTTAGATCTAACACCTCAAGTTGAATATGCAGCCTTGCAGACTGTT
GTGTATCCTGATAAGATAGTTAATGCTTTCTTCGGTCCGATCATAAAGGAAATTAATGAACGGATTATCAGAGCG
CTTAGACCTCATGTGGTCTTTAATTCTCGTATGACTGCTGATGAACTGAATGAAACAGTTGCCTTTTTGACACCT
CACAAGTACAGAGCCTTAGAGATTGACTTTTCAAAATTTGATAAATCAAAGACTGGGCTTCATATCAAAGCTGTC
ATTGGACTCTACAAGCTCTTTGGTCTAGATGGCCTGTTGAAAGTACTCTGGGAGAAATCGCAATTTCAGACTTAC
GTGAAAGATAGAAACTTCGGTCTCGAAGCATATCTATTATATCAGCAAAAGTCAGGAAACTGTGACACTTACGGT
TCGAACACCTGGTCTGCCGCCTTGGCGCTGTTAGATTGTCTTCCTTTGGAAGATGCGCATTTCTGTGTTTTTGGT
GGTGATGATTCATTGATATTGTTTGATCAGGGATACATAATATCCGACCCATGCCGGCAACTTGCCGGTACTTGG
AACCTTGAATGTAAAGTGTTCGACTTTAAGTACCCCGCATTTTGTGGTAAATTTCTGCTGTGTATCGATGGAAAA
TATCAGTTTGTTCCAGATGCGGCAAAGTTTATCACAAAATTAGGTAGAACTGATGTGAGAGATGTAGAAGTTTTG
AGTGAGATTTATATCTCTATCAATGACAATTACAAATCTTACAAAGACTTTAAGGTACTTGATGCTTTGGATAAG
GCTTTAGTGGACAGATATCGATCCCCTTATAGTGCTATTTCTGCTTTGGTTTCTTTGTGTTATCATATCTTTGAC
TTTAATAAGTTTAAGTTGCTGTTTAATTGTGAAGGGAAATTTGTGGATAAGAAGCTGAGAAAGGACTTCGAGTGG
TGAACTCTAGGGCCTGATGTTTAAATCTATTGTATTTACCTTCGCATGATGGCTACTTTCTCTTGTGTGTGTTGT
GGTACCTTAACTtggagcacTTATTGTGGTAAGAGATGTGAGCGAAAGCATGTATATTCTGAAACGAGAAATAAA
AGATTGGAACTTTACAAGAAGTATCTATTGGAACCGCAAAAATGCGCCCTGAATGAAATCGTTGGACACAGTTGT
GGAATGCTATGCTCCATTGCGGAAGAGGCTTGTGATCAGCTGCCAATCGTGAGTAAGTTCTGTGGCCAAAAGCAT
GCGGATCTGTATGATTTACTTCTGAAACGTTCTGAGCAGGAGTTACTTCTTGAATTTCTCCAGAAGAAGATGCAA
GAGCTGAAACTTTCTCATATCGTGAAGATGGCTAAGCTTGAAAGTGAGGTTAACGCAATACGTGAGTCCataggT
TCTTCTTGTGAAGATTCTGTTGGATGTGATGACTCTTCTTCCGTTTCTAAGTTGTAAAAAAAAAAAAAAAAAAAA
TGTTTGATCAGATCATTCAAATCTGATGGTGCCCATCAACCATATGATGGGAGTGTTTACAAGTCCACTATAATC
GAACTTGAAAACCTTGCCTGAATTGGAAACCATGAATCTTAACGGATTCTGGAGAGAAAATTTAGGAATTGGTAT
GTAAGCTACAACTTCCGGTAGCTGCGTCACACTTTAAGAGTGTGCATACTGAGCCGAAGCTCAGCTTCGGTCCCC
CAAGGGAAGACCA
